# Behavioral and Neural Dynamics of Interpersonal Synchrony Between Performing Musicians: A Wireless EEG Hyperscanning Study

**DOI:** 10.3389/fnhum.2021.717810

**Published:** 2021-09-13

**Authors:** Anna Zamm, Caroline Palmer, Anna-Katharina R. Bauer, Martin G. Bleichner, Alexander P. Demos, Stefan Debener

**Affiliations:** ^1^Sequence Production Laboratory, Department of Psychology, McGill University, Montreal, QC, Canada; ^2^Neuropsychology Laboratory, Institute for Psychology, European Medical School, University of Oldenburg, Oldenburg, Germany; ^3^Cluster of Excellence Hearing4All Oldenburg, University of Oldenburg, Oldenburg, Germany

**Keywords:** interpersonal synchrony, temporal coordination, neural entrainment, wireless electroencephalography, hyperscanning

## Abstract

Interpersonal synchrony refers to the temporal coordination of actions between individuals and is a common feature of social behaviors, from team sport to ensemble music performance. Interpersonal synchrony of many rhythmic (periodic) behaviors displays dynamics of coupled biological oscillators. The current study addresses oscillatory dynamics on the levels of brain and behavior between music duet partners performing at spontaneous (uncued) rates. Wireless EEG was measured from *N* = 20 pairs of pianists as they performed a melody first in Solo performance (at their spontaneous rate of performance), and then in Duet performances at each partner’s spontaneous rate. Influences of partners’ spontaneous rates on interpersonal synchrony were assessed by correlating differences in partners’ spontaneous rates of Solo performance with Duet tone onset asynchronies. Coupling between partners’ neural oscillations was assessed by correlating amplitude envelope fluctuations of cortical oscillations at the Duet performance frequency between observed partners and between surrogate (re-paired) partners, who performed the same melody but at different times. Duet synchronization was influenced by partners’ spontaneous rates in Solo performance. The size and direction of the difference in partners’ spontaneous rates were mirrored in the size and direction of the Duet asynchronies. Moreover, observed Duet partners showed greater inter-brain correlations of oscillatory amplitude fluctuations than did surrogate partners, suggesting that performing in synchrony with a musical partner is reflected in coupled cortical dynamics at the performance frequency. The current study provides evidence that dynamics of oscillator coupling are reflected in both behavioral and neural measures of temporal coordination during musical joint action.

## Introduction

Many joint actions, from ensemble music performance to team rowing, require that partners synchronize the timing of rhythmic (oscillatory) movements. There is growing evidence that interpersonal synchrony of rhythmic movements is influenced by dynamics of entrainment between biological oscillators (Kelso, 1984). An external rhythmic signal such as music may evoke intrinsic neural oscillations that entrain to the periodicities in the rhythmic sequence. Entrainment occurs when two oscillating systems, which have different periods when they function independently, assume the same period (or integer-ratio related periods) when they interact (Pikovsky et al., 2003). Mathematical entrainment between oscillators is constrained by differences in the intrinsic frequencies of the oscillators, combined with their period coupling (Haken et al., 1985; Strogatz, 2003; Kuramoto, 2012). Coupled oscillators with similar natural frequencies will show greater entrainment than will coupled oscillators with different frequencies (Haken et al., 1985).

Evidence for principles of oscillator entrainment in interpersonal coordination comes from a growing body of behavioral research. Much of this work is based on ‘frequency detuning’ paradigms from studies of intra-limb coordination (Turvey, 1990) in which partners coordinate the swinging of hand-held pendulums or swaying of rocking chairs that are weighted to have either the same or different frequencies of motion. The synchronization of partners’ movements is a function of the difference in their rocking chair or pendulum frequencies (Schmidt and O’Brien, 1997; Richardson et al., 2005, 2007; Lopresti-Goodman et al., 2008).

Music performance studies have directly tested the hypothesis that partners’ *spontaneous frequencies*—unconstrained frequencies of self-paced spontaneous rhythmic motion during music performance—influence interpersonal entrainment (Loehr and Palmer, 2011; Zamm et al., 2015, 2016; Palmer et al., 2019). These studies showed that differences in partners’ spontaneous rates of solo music performance predicted synchronization accuracy and precision during duet performance paced by a metronome cue. Moreover, smaller differences in partners’ spontaneous frequencies were associated with greater duet synchronization accuracy and precision relative to larger differences in spontaneous frequencies.

Evidence to suggest that spontaneous rates of music performance reflect stable intrinsic frequencies comes from several findings. First, individuals who differ in skill, musical preferences, and experience on different instruments have been documented to have consistent spontaneous performance tempi. These consistent individual differences are shown not only in the mean rate but also in the variance (Zamm et al., 2019), with lowest temporal variance at the spontaneous rate and higher variance at both slower and faster rates than the individuals’ spontaneous rate. Second, the size and direction of differences in duet partners’ spontaneous rates are predictive of how well they synchronize, consistent with the mathematical prediction of similar oscillator frequencies generating greater synchronization (Zamm et al., 2016; Palmer et al., 2019). This finding has been replicated in both music performance and tapping tasks, with musicians and non-musicians (Scheurich et al., 2018). Third, the spontaneous rates are consistent within individuals, across their limb movements and melodies (Zamm et al., 2016), across time of day (Zamm et al., 2019; Wright and Palmer, 2020). Moreover, recent computational work (Roman et al., 2020) indicates that the relationship between musical partners’ spontaneous frequencies and interpersonal synchrony can be accurately predicted from a model of biological oscillator entrainment, providing further credence to an oscillator framework of musical synchrony.

Oscillator dynamics are also reflected in electrophysiological brain measures. Electroencephalography (EEG) research has shown that neural oscillations entrain to the frequency of external rhythms (Galambos et al., 1981; Tobimatsu et al., 1999; Ross et al., 2003; Feurra et al., 2011; Bauer et al., 2020). Neural entrainment has been characterized by enhanced spectral energy of EEG oscillations at the stimulus frequency relative to other frequencies, and by phase alignment of EEG oscillations with the stimulus phase (see Lakatos et al., 2019 for a review). Neural entrainment to external rhythms not only supports the ability to accurately perceive rhythms (Henry et al., 2015; Bauer et al., 2018), but also to coordinate the timing of movements with those auditory rhythms (Nozaradan et al., 2013, 2016). Specifically, greater spectral energy of EEG oscillations at the frequency of an external stimulus has been associated with smaller asynchronies between movement and stimulus (Nozaradan et al., 2016).

It has been suggested that partners’ neural activity becomes coupled during temporal coordination in joint action (Tomlin et al., 2006; Tognoli et al., 2007; Dumas et al., 2010; Astolfi et al., 2011; Funane et al., 2011; Cui et al., 2012; Müller and Lindenberger, 2014; Lee, 2015). Lindenberger and colleagues showed that guitarists’ duet synchronization is accompanied by inter-brain phase coupling of oscillations in the delta frequency range (Lindenberger et al., 2009; Sänger et al., 2012). Novembre et al. (2017) demonstrated that inducing inter-brain coupling through in-phase transcranial alternating current stimulation leads to enhanced interpersonal coordination of dyadic finger tapping relative to anti-phase stimulation. It is unknown how period coupling is reflected in inter-brain correspondences. However, prior work does suggest that period coupling of one’s behaviors with external auditory rhythms is associated with enhanced amplitude of cortical oscillations at the coupling frequency relative to other frequencies (Nozaradan et al., 2013, 2016). Thus, we hypothesize that production of a common frequency during duet performance is accompanied by enhanced spectral energy of partners’ neural oscillations at the common frequency of partners’ performances relative to other frequencies; moreover, we predict that the amplitude envelopes of oscillations at the common frequency are coupled between partners, reflecting co-production of a shared rhythmic structure.

Amplitude envelopes, defined as the absolute value of the Hilbert transform of a neural oscillation, reflect energy fluctuations over time (Bruns et al., 2000). Correlations between the amplitude envelopes of two brain signals measure the degree to which the amplitude fluctuations are temporally correlated. Amplitude envelope correlations (AECs) have been used to detect functional connectivity, that is, synchrony between functional brain networks, both within and across frequency bands (Bruns et al., 2000; Hipp et al., 2012; Zamm et al., 2018). AECs are sensitive to long-range dependencies (Bruns et al., 2000), and MEG studies found that they show superior test-retest reliability relative to other standard functional connectivity metrics (e.g., phase- or coherence-based metrics; Colclough et al., 2016). Moreover, amplitude envelope metrics are less susceptible to measurement jitter than alternative phase-based metrics, an important consideration for naturalistic music performance conditions. The sensitivity of AECs for detecting inter-brain correspondences between performing musicians has been demonstrated with wireless EEG (Zamm et al., 2018). We apply AECs here to assess inter-brain correspondences at the performance rate of duet performances.

The current study investigated oscillator dynamics of interpersonal synchrony in duet music performance by investigating the extent to which partners’ spontaneous performance rates influence interpersonal synchrony during temporally unconstrained performance. We also investigated whether partners display inter-brain coupling of cortical oscillations at the duet performance rate. Duet music performance was used as a model of oscillatory joint action, since music is highly rhythmic and musicians must be able to coordinate production of these rhythms with millisecond precision. We randomly paired pianists and recorded wireless EEG while pianists performed a simple melody in the Solo condition at their spontaneous rate and in Duet performance with their partner. In Duets, partners took turns as Leader to set the pace of performance at their spontaneous rate, in contrast with much of the previous literature on coordinate dynamics of music performance, where musicians produce actions at a cued rate. The use of wireless EEG enabled us to investigate inter-brain synchrony during music performance with minimal artifact (Debener et al., 2012; De Vos et al., 2014a, b). Moreover, use of wireless EEG allowed musicians to move freely and naturally as wireless EEG has been shown to be feasible in mobile conditions (e.g., Scanlon et al., 2020).

We hypothesized that interpersonal coordination in duet music performance is constrained by the amount of coupling needed to overcome differences in partners’ natural frequencies. Based on previous findings, Duet tone onset synchronization was predicted to decrease as a function of the difference in partners’ spontaneous rates (Loehr and Palmer, 2011; Zamm et al., 2015; Palmer et al., 2019). Larger tone onset asynchronies (Leader − Follower tone onsets) were expected between duet partners with larger differences in spontaneous frequency relative to partners with smaller differences. Furthermore, we hypothesized that frequency coupling is reflected on a neural level during Duet performance. Specifically, we predicted that partners’ neural oscillations show increased power at the performance rate of tone production during Duets, reflected as higher spectral amplitude of EEG signals at the Duet frequency relative to other frequencies. Furthermore, higher spectral amplitudes at the performance rate should be positively associated with Duet tone onset synchronization. Finally, partners’ amplitude envelopes of neural oscillations should be correlated at the common frequency of Duet music performance, yielding inter-brain correspondences.

## Materials and Methods

### Participants

40 pianists were randomly assigned to 20 pairs (25 female, mean age = 23.96 years, range = 18–40) with six or more years of private piano lessons (mean = 11.16 years, range = 6–18) were recruited from the Oldenburg community. Pianists were included if they had self-reported normal or corrected to normal vision, no current psychiatric or neurological conditions or use of neuropsychiatric medication, self-reported normal hearing confirmed through a pure-tone audiometric screening test (< 20 dB binaurally for the range of tones in the stimulus melody) and right-hand dominance. Handedness was confirmed using the Edinburgh Handedness Inventory (Oldfield, 1971). All participants scored as right-hand dominant (*M* = 85.9, *SD* = 17.2), except one individual who showed a tendency toward left-hand dominance (score = 36.8). The study protocol was reviewed by the local ethics committees at the University of Oldenburg and McGill University, and all participants provided informed consent prior to recording, in accordance with the Declaration of Helsinki. EEG data associated with Solo performances from the current sample have been reported in Zamm et al. (2019) to demonstrate the validity of wireless EEG for measuring the neural correlates of music performance; these EEG data are not reported here. EEG data associated with Duet performances from 2 pairs from the current sample have been reported in Zamm et al. (2018) to demonstrate the validity of amplitude envelope correlations for measuring inter-brain correspondences in oscillatory activity.

### Materials

Participants performed the popular melody *Bruder Jakob* (Frère Jacques, Brother John) on the piano with their right hand. Participants were familiar with the melody and rehearsed the melody from stimulus notation sent in advance of their arrival at the laboratory; the melody was notated in C major (treble clef) and the rhythm was in binary (4/4) meter and was 32 tones in duration (20 quarter notes, 4 half notes, 8 eighth notes; note range = G3–A4). To control for possible differences in finger movements across pianists, suggested fingerings were indicated on the melody notation (provided by three skilled pianists not in the study).

### Equipment

#### Keystroke Recording

**FIGURE 1:**
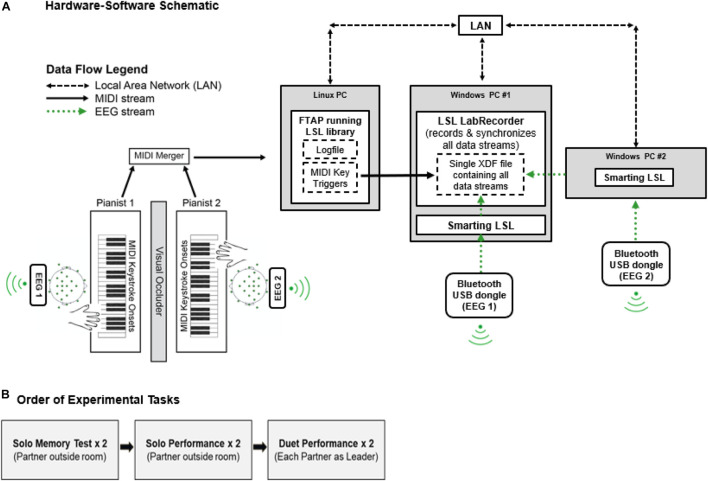
**(A)** Hardware-software schematic. MIDI keystroke data (solid black arrows) from duet pianists are merged and sent to FTAP software running the LSL library in a Linux OS. LSL sends MIDI time-stamps over the Local Area Network (LAN, dashed black arrows) to LabRecorder software, where timestamps are recorded as keystroke event triggers. EEG data streams (dotted green arrows) from duet pianists are sent *via* Bluetooth to SmartingLSL software running the LSL Library in a Windows OS. LSL sends EEG data streams to LabRecorder software. LabRecorder software synchronizes MIDI and EEG data streams by correcting for clock offsets between respective data acquisition computers (Linux/Windows). Synchronized data streams are stored in a single XDF file for storage. **(B)** Order of Experimental Tasks.

Figure 1 shows the Duet experiment set-up. Two identical Yamaha P35B Musical Instrument Digital Interface (MIDI) DC-powered keyboards (Yamaha Corporation, Japan) were used to record duet piano performances. The keyboards were positioned to face one another in the testing room. A shoulder-level occluder was placed between the keyboards to minimize possible influences of visual cues from a partner’s finger movements on duet partners’ temporal coordination. Auditory feedback associated with pianists’ keystrokes was presented through speakers on each keyboard, and volume was calibrated to equal loudness across keyboards using a sound meter. MIDI information (timestamps, pitch, velocity) from pianists’ keystrokes was sent on two separate channels, merged *via* a MIDI-USB merger (Prodipe Corporation, France), and sent to a Linux (Fedora) operating system computer running FTAP MIDI recording software (Finney, 2001). The Linux computer was connected to the local area network (LAN) *via* an ethernet switch (TP-Lin GmbH). FTAP was adapted to include the Lab Streaming Layer library (LSL; Kothe, 2014^1^), which allowed for sending MIDI keystroke triggers to LabRecorder software (v1.1)^2^) over the LAN.

#### EEG Recording

Two 24-channel mobile EEG systems (SMARTING mBrain Train LLC^3^), attached to separate elastic electrode caps^4^, were used to record EEG data from participants. Electrode positioning followed the international 10–20 system. FCz served as the reference electrode and AFz served as the ground electrode. Electrode impedances were below 20 kOhms before the recording started. Wireless EEG amplifiers (weight = 60 g; size = 82 mm × 51 mm × 12 mm; resolution = 24 bits; sampling rate = 500 Hz) were strapped to the back of each participant’s electrode cap between O1 and O2 electrode sites. Digitized EEG data from each participant were sent wirelessly from the amplifier to a Bluetooth receiver placed on the wall directly behind their keyboard. Bluetooth receivers sent this information *via* USB to Windows 7 computers running SmartingLSL, which collected the data using the LSL library and sent the data to LabRecorder over the LAN. LabRecorder synchronized EEG data from both amplifiers with MIDI data by correcting for clock offsets between acquisition computers. For details on synchronizing MIDI and EEG data from a single mobile amplifier, it was confirmed that MIDI and EEG recordings were synchronized with millisecond precision (Zamm et al., 2019). Synchronization between two EEG amplifiers and two MIDI keyboards was confirmed through timing tests which are available upon request.

### Experimental Design

Each pianist performed two tasks with the same stimulus melody: a Solo task and a Duet task. The Solo task always preceded the Duet task to ensure that each partner’s spontaneous rate (acquired during Solo performance) was not influenced by their partner’s performances. The Solo Task measured each pianist’s spontaneous rate on 3 trials, where 1 trial included 4 continuous performances of the melody (yielding 12 repetitions per pianist). Each pianist completed 1 practice trial and 3 test trials of Solo performance, yielding a total of 12 Solo test performances of the melody.

The Duet task measured the pianists’ tone onset asynchrony while one partner played the role of Leader (partner responsible for cueing the pace of the Duet performance) and the other partner played the role of Follower (partner responsible for performing at the Leader’s pace) as they tried to synchronize their performances. Two independent variables were manipulated in a within-subject design. The first independent variable, Musical Role (Leader, Follower) was coded for analyses that examined each pianist as the random variable. The second independent variable, Leader-Order condition, included 2 levels (First-Leader, Second-Leader) and was coded for analyses that examined each pair as the random variable. In the First-Leader condition, the duet partners chose which partner would play the role of Leader. In the Second-Leader condition, the experimenter assigned the role of Leader to the partner who played the role of Follower in the First-Leader condition. Each Leader-Order condition comprised 1 practice trial and 6 test trials of duet performance (1 trial = 4 continuous melody repetitions), yielding a total of 24 Duet test performances of the melody for each of the two conditions (First-Leader, Second-Leader).

#### Task and Procedure

Pianists were sent the melody’s stimulus notation in advance of the study, with the instruction to practice with the notated fingering until memorized. When Duet partners arrived at the lab, they were each given a short practice session and then separately completed a melody memory test, as shown in Figure 1B. In the memory test, each partner was instructed to perform the melody without pitch or rhythm errors. While one partner completed the memory test, the other partner waited outside of the testing room. Each partner was given up to two attempts at passing the memory test or else were excluded from the study. All participants passed the memory test after two attempts. After passing the melody memory test, the pianists completed two performance tasks, a Solo task and a Duet task. In the Solo performance task, each partner performed the melody alone with the instruction to produce tone onsets at a regular rate that felt natural and comfortable for them. While one partner completed the Solo performance task, the other partner waited outside of the testing room where they could not hear their partner’s performance.

Partners subsequently completed the Duet performance task, in which partners performed the melody together simultaneously. One partner (the Leader) was instructed to set the pace of each performance at a rate that was natural and comfortable for them just as they did during Solo performance, while their partner (the Follower) was instructed to follow the Leader’s rate and to synchronize their production of tone onsets with the Leader’s. In the First-Leader condition, Duet partners decided amongst themselves who would be the Leader and who would be the Follower. Partners performed 6 test trials of the First-Leader condition. Then, in the Second-Leader condition, partners switched roles and the First Leader became the Follower while their partner became the Leader, and 6 test trials were subsequently performed in this condition. On each trial of the Duet performance, the partner assigned as Leader cued the performance rate by performing the first 8 melody tones alone, allowing the Leader to clearly signal the performance tempo to their partner at the beat level (defined by the first 8 tones which were all quarter notes, by comparison to the rest of the melody which featured a combination of quarter-, eighth-, and half-notes). The Follower joined the Leader on the 9th melody tone, and the rest of the trial was played entirely in unison (the two pianists intended to produce the same pitches at the same time). EEG was recorded during Solo and Duet piano performance tasks. Resting state EEG data was collected from both partners, as was EEG data associated with a second Duet task (after the Second-Leader condition) in which partners performed the melody cued by a metronome and there was no Leader; these data are not reported in the current paper.

## Analyses

### Data Cleaning

#### Identification and Removal of MIDI Keystroke Errors

Since pitch errors in music performance often coincide with timing errors (Drake and Palmer, 2000), melody repetitions containing pitch errors (defined as added or deleted tones) were identified using the MIDI Matcher program in MATLAB (v1.1; Large, 1993) and were excluded from both behavioral and neural analyses (rarely occurring substituted pitches were retained as these do not disrupt the timing of pitch sequencing). For Solo performances, this procedure resulted in exclusion of 3.3% of the total melody repetitions (16/480 melody repetitions; see Zamm et al., 2019). For Duet performances, 3.96% of the total melody repetitions (38/960 melody repetitions) were excluded.

#### EEG Artifact Correction

EEG data were corrected for artifacts using EEGLAB (Delorme and Makeig, 2004). First, data were concatenated across all trials and conditions in the entire study, filtered between 1 and 40 Hz with a Hanning windowed sinc FIR filter (low pass order = 100; high pass order = 500; Widmann and Schröger, 2012), segmented into consecutive 1 s epochs, and pruned for non-stereotypical artifacts (kurtosis limit = 2). Data were then submitted to extended infomax Independent Component Analysis (ICA; Bell and Sejnowski, 1995; Jung et al., 2000a, b). ICA components that reflected eye blinks, lateral eye movements and other sources of non-cerebral activity were identified and removed from the data. This procedure resulted in the removal of 1–5 components per subject (*M* = 2.6, *SD* = 0.98). Artifact-corrected data were referenced to the common average across electrode sites. A single bad channel was identified in a single participant, and replaced by means of spherical interpolation as implemented in EEGLAB.

### Definition of Analysis Window

All behavioral and EEG spectral density measures were computed over a fixed-duration analysis window. The use of a fixed duration window was necessary for spectral density analyses to ensure identical frequency resolution across participants’ spectra; behavioral data were analyzed over the same window to ensure that neural and behavioral comparisons were made across the same data segments.

The window duration of 9 s was selected, corresponding to the fastest pair’s mean duration of melody performances across Duet conditions (2.4% of total melody performances in the sample were faster than this duration). This window was defined relative to the fastest pair (and not the slowest pair) so that padding was not necessary to achieve equivalent window duration across performances. Neural oscillations at the duet frequency were assumed to show slow (low-frequency) changes over time; therefore for this analysis only we assessed amplitude envelope measures over the full melody performance instead of the shorter 9-second window to allow sufficient time for capturing their temporal dynamics.

### Behavioral Measures of Temporal Coordination

#### Performance Rates

Mean Solo and Duet Performance rates were defined as the mean inter-tone onset interval (IOI, milliseconds) at the quarter-note level across each participant’s melody performances (half notes were linearly interpolated prior to IOI computation and off-beat eighth notes were excluded, consistent with rate calculations in previous studies of piano duet performance; Loehr and Palmer, 2011; Zamm et al., 2015, 2016). Mean IOIs for Duet performances were first computed separately for the Leader and Follower. Leaders’ and Followers’ mean IOIs did not differ significantly in either Leader-order condition, First-Leader: *t*(38) = 0.015, *p* = 0.99; Second-Leader: *t*(38) = 0.016, *p* = *0.99*. Therefore, Leaders’ and Followers’ mean Duet performance IOIs were averaged to yield a single mean Duet IOI for each pair in each Duet condition. To facilitate comparison with EEG frequency spectra, mean Solo and Duet IOIs were converted to Hertz (Hz = 1,000/mean Duet IOI in ms, or # tones/s). Whereas Zamm et al. (2019) converted Solo IOIs to Hz prior to averaging the means (for comparison with EEG measures), the current manuscript changed the method slightly to allow for comparisons between behavioral measures of Solo performance rates with Duet asynchronies, which are computed in milliseconds.

#### Duet Synchronization

Two well-established measures of tone onset asynchrony were computed for each Duet pair on their Duet performances (Repp and Su, 2013): signed asynchrony and absolute asynchrony. Signed asynchrony was defined as the mean signed difference in piano keystroke onset times that partners intended to perform simultaneously (Leader’s onsets − Follower’s onsets). Signed asynchrony provides a measure of Leading-Following behavior, where negative values indicate that the Leader’s tone onsets preceded the Follower’s. Signed duet asynchronies permit tests of the hypothesis that Leading-Following behavior in Duet performance is a function of how much faster the Leader’s spontaneous (Solo) rate is relative to the Follower’s. Absolute asynchrony was defined as the absolute difference between tone onsets that partners intended to perform simultaneously [abs (Leader’s onsets − Follower’s onsets)]. Absolute asynchrony provides a measure of a given pair’s overall synchronization accuracy, where small asynchrony values indicate high synchronization accuracy. Absolute asynchronies permit tests of the hypothesis that overall synchronization accuracy between performing musicians is associated with the degree to which they showed neural entrainment at the Duet Performance Rate. Both signed and absolute synchronies for each performance were computed as a proportion of the mean Duet IOI for that performance, to adjust for differences in performance rate across pairs.

### EEG Measures of Neural Entrainment

#### Power Spectral Density of Oscillations at Duet Performance Rate

Neural entrainment during Duet performance was defined within each duet condition by the EEG Power Spectral Density (PSD) of each partner, computed at the pair’s mean Duet Performance Rate (section “Performance Rates”). This definition is consistent with previous work defining stimulus entrainment by enhanced spectral amplitude of oscillations at the stimulus frequency (Nozaradan et al., 2011, 2012, 2013). The mean Duet Performance Rate was computed as the mean IOI across partners and across melody performances within each Leader-Order condition (*n* = 24 in cases for which no performances were discarded due to errors). PSD was computed separately for each melody repetition and then averaged to obtain a single value per partner, per Leader-Order condition.

To compute PSD, artifact-corrected EEG data were low-pass filtered (Hanning windowed sinc FIR filter, 20 Hz, order 1,000), high-pass filtered (0.1 Hz, order 1,000), and epoched into 9 s segments (for the 2.4% of melody repetitions shorter than 9 s, epochs extended into the beginning of the subsequent melody repetition). To reduce edge effects, epochs were multiplied with a 4,500-point (9 s) Hanning window, and the PSD was computed for each epoch and channel using Welch’s method (*pwelch* function in MATLAB; frequency resolution = 0.061 Hz). Resulting power spectra were log-transformed to compensate for 1/*f* power distribution characteristic of EEG data, and then separately averaged across epochs on each channel for each participant.

A noise normalization procedure was subsequently applied by subtracting from each frequency bin the mean power at ± 3 neighboring frequency (± 0.183 Hz) bins (Nozaradan et al., 2011, 2012; Tierney and Kraus, 2013, 2014; Zamm et al., 2019). This procedure should result in cancellation of noise-related spectral peaks and preserve spectral peaks associated with non-noise components. Noise-subtracted mean spectra were subsequently computed for each participant by averaging noise-subtracted spectra across all electrode sites. Mean spectra at a fronto-central Region of Interest (ROI = FC1, FC2, Cz, and Fz) were also computed for each participant, based on previous findings (Zamm et al., 2019) that identified this ROI as showing maximal power at the frequency of the self-paced Solo piano performances. To allow for comparison of spectral power at each pair’s unique mean Duet Performance Rate for each condition, power spectra were aligned across participants using a 2.99 Hz window centered on their mean Duet Performance Rate in Hertz (corresponding to ± 24 frequency bins on either side of the Duet Performance Rate, the number of bins between the slowest mean Duet Performance Rate and the lower edge of the frequency spectrum after noise subtraction).

#### Amplitude Envelopes of EEG Oscillations at the Duet Performance Rate

##### Calculation of Amplitude Envelopes

Amplitude envelopes of EEG oscillations at the Duet performance rate were computed to assess the dynamics of the Duet partners’ neural responses over time using methods described in detail in Zamm et al. (2018). First, pianists’ EEG data in each Duet condition were spatially filtered to extract a single time-course of oscillations at their unique Duet performance rate. Spatial filters were implemented to improve the signal-to-noise ratio associated with neural oscillations at the Duet performance rate while also reducing multiple comparison issues associated with multi-dimensional EEG data. Spatial filters were obtained by submitting each pianist’s Solo EEG data—representing an independent data set for the same pianists from a comparable task—to spatio-spectral decomposition (SSD), a linear decomposition algorithm tailored for extracting oscillations in a target frequency band while attenuating neighboring frequencies (Nikulin et al., 2011; Dähne et al., 2014). SSD was computed to extract oscillations in the range of observed Solo performance rates (1.5–3.5 Hz), corresponding closely to the range of observed Duet rates. For each participant, the first and final authors visually selected the SSD spatial filter most clearly representing a stereotypical fronto-central auditory-motor delta topography. After selecting spatial filters for each pianist, each pianist’s artifact-corrected Duet EEG data in each condition were filtered around their Duet performance rate (mean Duet rate ± 0.183 Hz, signal bandwidth = 0.366 Hz, 2nd order butterworth filter), and multiplied with their selected spatial filter, yielding a single time course of neural oscillations at the Duet performance rate. This time course was epoched into segments corresponding to the duration of each melody repetition ± 2.5 seconds (s) and down-sampled to 100 Hz using an antialiasing FIR filter (pop_resample.m in EEGLAB), which improved the efficiency of subsequent calculations while remaining significantly above the Nyquist criterion. Amplitude envelopes were subsequently defined as the absolute value of the Hilbert transform of each melody epoch. To minimize edge artifacts of the Hilbert transform, 2.5 s tails were trimmed. Zamm et al. (2018) reports further detail on the SSD decomposition, including topographic maps.

Because musicians do not perform the same melody with identical timing across the tone sequence (Palmer, 2013), the number of EEG samples between corresponding melody tones differed across performances. To allow for comparison across performances within each pair and Duet condition, partner’s amplitude envelopes were resampled such that the number of samples between corresponding tone events was constant across different performances of the stimulus melody for each pair and condition. First, the minimum number of samples between tone onsets was determined across melody repetitions within the pair and condition. This minimum value was used to resample all IOIs. The number of samples for each eighth-note interval (the shortest notated IOI) was set equal to this number. Quarter notes contained twice this number, whereas half notes contained 4 times this number. Shape-preserving piece-wise cubic interpolation (interp1.m in MATLAB, using “pchip” and “extrap” arguments), which fits a cubic polynomial between each set of interpolation points, was applied to preserve the original shape of the resampled signal. Thus, amplitude envelopes could be averaged across melody repetitions while the data segments being averaged corresponded to the same tone onsets.

##### Inter-Brain Correlations of Duet Partners’ Amplitude Envelopes

To quantify correspondences in the amplitude dynamics of partners’ neural oscillations at the Duet performance rate, inter-brain correlations of EEG amplitude envelopes (Amplitude Envelope Correlations, AECs) were assessed for each pair, using the method described in Zamm et al. (2018). Specifically, AECs were computed for each melody repetition within each pair and Leader-order condition. For the first melody repetition in each trial, AECs were computed over data occurring after the 8th tone (during which both partners were performing). Inter-brain AECs for each melody repetition were subsequently converted using the Fisher-*z* transform to ensure normality, and averaged across melody repetitions, within-pair and within-condition.

To test whether observed inter-brain AECs reflected amplitude correspondences specific to each Duet pair, AECs from observed Duet pairs were compared with AECs of surrogate pairs. Surrogate pairs were created within each condition by pairing each Leader with all Followers except their true partner: This procedure yielded 19 surrogate Duet pairs per Leader and per condition. For each surrogate pairing, amplitude envelopes were resampled within Duet condition such that the number of samples between corresponding tone events was constant across melody repetitions, using the same procedure described above. Resampling was necessary because surrogate partners’ performances occurred at different rates, and their data could not be compared without resampling relative to the musical event structure. After resampling, amplitude envelopes of corresponding melody repetitions in the trial structure were correlated (24 repetitions per condition), and averaged using the same procedure as for observed pairs. Amplitude envelope correlations were averaged within each surrogate pair across conditions.

## Results

### Behavioral Measures

#### Solo Performance Rates

**FIGURE 2:**
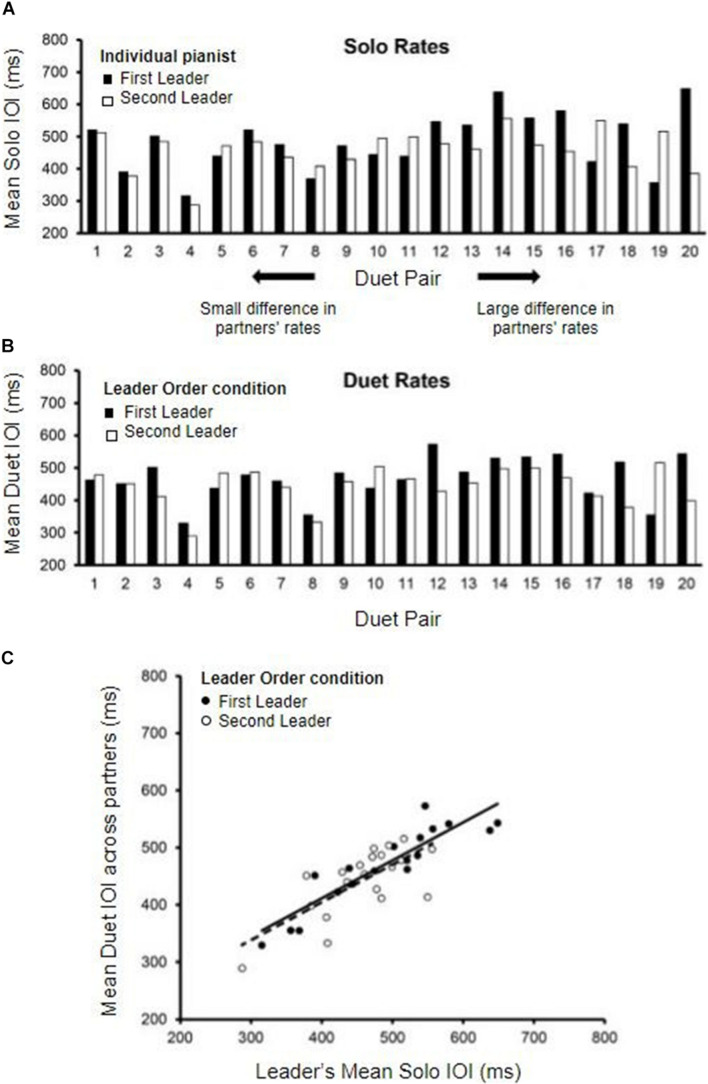
Solo and Duet performance rates. **(A)** Mean Solo performance rates for individual partners; partners are labeled by who served as Leader in the First-Leader (black bars) and Second-Leader (white bars) Duet conditions. Pairs are ordered by the magnitude of difference in partners’ Solo rates (smallest difference on the left, largest on the right). **(B)** Mean Duet performance rates for each pair (averaged across partners) in the First-Leader (black bars) and Second-Leader (white bars) conditions. Pairs are ordered the same as in panel **(A)**, by the magnitude of difference in partners’ Solo rates. **(C)** Leaders’ mean Solo performance rates (x-axis) correlated with Mean Duet performance rates (y-axis) in the First-Leader (black dots) and Second-Leader (white dots) conditions, with trend lines (solid = First-Leader condition; dashed = Second-Leader condition). Each dot represents data from one pair.

The distribution of individual pianists’ Solo performance rates (mean IOI in ms) is shown in Figure 2A. The data are displayed by Duet pair, to show the range of differences among randomly assigned partners. Solo rates for each pair are ordered from smallest difference between partners to largest difference. A range of Solo performance rates was observed across participants, with nearly doubled rate increase from the fastest pair average (302 ms) to the slowest pair average (597 ms).

The differences between Duet partners’ Solo performance rates were examined in terms of the Leader-Order Duet conditions, to determine whether one Leader-Order condition differed from the other for the randomly paired partners. The mean difference in partners’ Solo performance rates was 74.26 ms (range = 8.6 to 263.76 ms, *SD* = 62.07 ms). The First and Second Leaders’ Solo rates did not differ significantly, *t*(19) = −1.29, *p* = 0.21 (First-Leader mean = 485.46 ms; Second-Leader mean = 458.23 ms), ensuring that any subsequently observed differences between First-Leader and Second-Leader conditions were not simply a function of their random assignment to Duet pairs.

#### Duet Performance Rates

We compared the mean Duet performance rates of Leaders and Followers within Leader-Order conditions. Those mean values indicated that the duet partners were performing at the same rate within condition, as expected [First-Leader means: *t*(38) = 0.015, *p* = 0.99; Second-Leader means: *t*(38) = 0.016, *p* = 0.99]. Therefore, the mean Duet performance rates within condition were defined for each Duet pair as the mean IOI averaged across the two duet partners. The distribution of mean Duet performance rates by Leader-Order condition is shown in Figure 2B, which orders pairs the same way as in 2A (by difference in Solo rates). As shown, the difference between First-Leader and Second-Leader mean Duet IOIs tended to be smaller for pairs with smaller differences in Solo rates (left end of Figures 2A,B) and larger for pairs with larger differences in Solo rates (right end of Figures 2A,B). Duet performance rates in each Leader-Order condition were then correlated with each partner’s Solo performance rate to determine whether the Leader set the rate of Duet performance to a value similar to their Solo rate. As shown in Figure 2C, the correlations of the Leader’s mean Duet performance rates with Leader’s Solo rate were significant, [*r* (18)*_*First–Leader*_* = 0.90, *r*(18)*_*Second–Leader*_* = 0.71, both *p*’s < 0.001], whereas the correlations with Follower’s Solo performance rate were not [*r*(18)*_*First–Leader*_* = 0.24, *p* = 0.30, *r*(18)*_*Second–Leader*_* = 0.31, *p* = 0.19].

To confirm that the Leader’s Solo rate had a greater influence on the Duet performance rates than did the Follower’s Solo rate, a multiple regression model predicting Duet performance rate from Leader’ Solo rate and Follower’s Solo rate was implemented separately for each Duet condition. For the First-Leader condition, this regression model yielded a significant fit, *R*^2^ = 0.81, *F*(2, 17) = 37.70, *p* < 0.001: The Leader’s Solo performance rate contributed significantly, standardized β = 0.908, *t*(17) = 8.36, *p* < 0.001, whereas the Follower’s Solo performance rate did not, standardized β = −0.02, *t(*17) = 0.15, *p* = 0.881. For the Second-Leader condition, this regression model also yielded a significant fit, *R*^2^ = 0.52, *F*(2, 17) = 9.22, *p* = 0.002: Again, the Leader’s Solo rate contributed significantly, standardized β = 0.68, *t*(17) = 3.88, *p* = 0.001, whereas the Follower’s rate did not, standardized β = 0.11, *t*(17) = 0.64, *p* = 0.53. These results confirm that Leaders in both conditions set the tempo of Duet performance close to their Solo performance rate, and Followers performed at the Leaders’ rate.

#### Duet Synchronization and Correlations With Solo Rates

Duet pairs’ mean signed asynchronies (Leader − Follower tone onsets, divided by mean Duet IOI) across Leader conditions ranged from negative to positive values (range = −0.056 to 0.113). Tests of Leader Order effects on signed asynchronies yielded no significant differences [*M*_*First–Leader*_ = 0.0047; *M*_*Second–Leader*_ = −0.0036, *t*(19) = −0.70, *p* = 0.49]. Duet pairs’ mean absolute asynchronies were also computed [abs (Leader − Follower tone onsets) divided by mean Duet IOI], and ranged from 0.033 to 0.124 across Leader conditions. Tests of Leader Order effects on absolute asynchrony indicated no significant differences [*M*_*First–Leader*_ = 0.050, *M*_*Second–Leader*_ = 0.054, *t*(19) = 1.12, *p* = 0.28], suggesting that overall synchronization accuracy was not significantly influenced by whether the partner served as Leader in First or Second Leader conditions.

**FIGURE 3:**
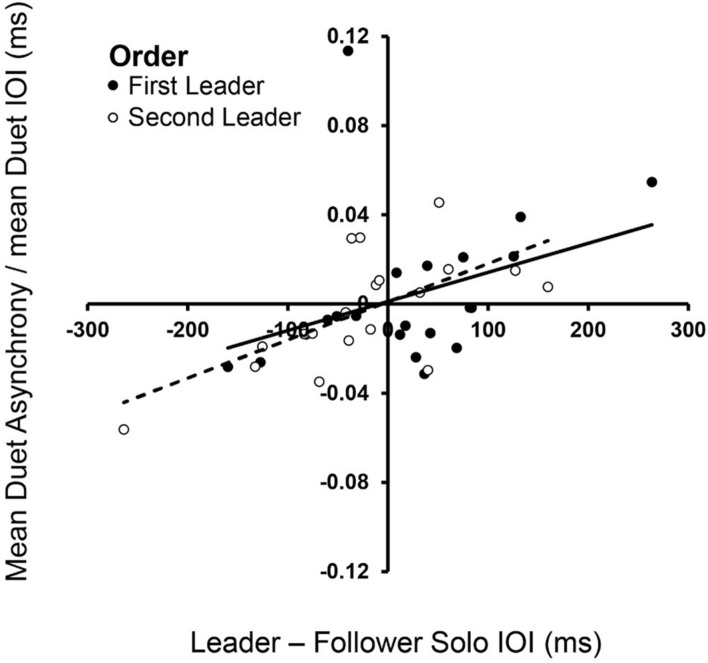
Spontaneous frequencies in Solo performance influence Duet synchronization. Signed differences in partners’ mean Solo rates (Leader Solo mean IOI—Follower Solo mean IOI, x-axis) correlated with signed Duet tone onset asynchronies (Leader tone onset—Follower tone onset, divided by mean Duet performance rate) in First-Leader (black dots) and Second-Leader (white dots) conditions. Each dot represents data from one pair.

Dynamical systems hypotheses predict that the signed asynchrony, which is the degree to which Leaders’ tone onsets preceded Follower’s tone onsets, is related to how much faster the Leader’s Solo rate is relative to the Follower’s (Zamm et al., 2015). We tested this hypothesis by determining whether the signed asynchrony was related to the difference in partners’ Solo performance rates, which ranged widely across Duet pairs. Figure 3 shows that the signed difference in partners’ Solo performance rates was significantly correlated with their signed Duet asynchronies in both Leader-Order conditions [First-Leader: *r*_*s*_(18) = 0.49, *p* = 0.028; Second-Leader: *r*_*s*_(18) = 0.65, *p* = 0.002], consistent with dynamical systems predictions that coupling between oscillators is a function of the difference in their natural frequencies.

### EEG Power Spectral Density

#### Channel-Mean PSD Peaks at Duet Performance Rate

**FIGURE 4:**
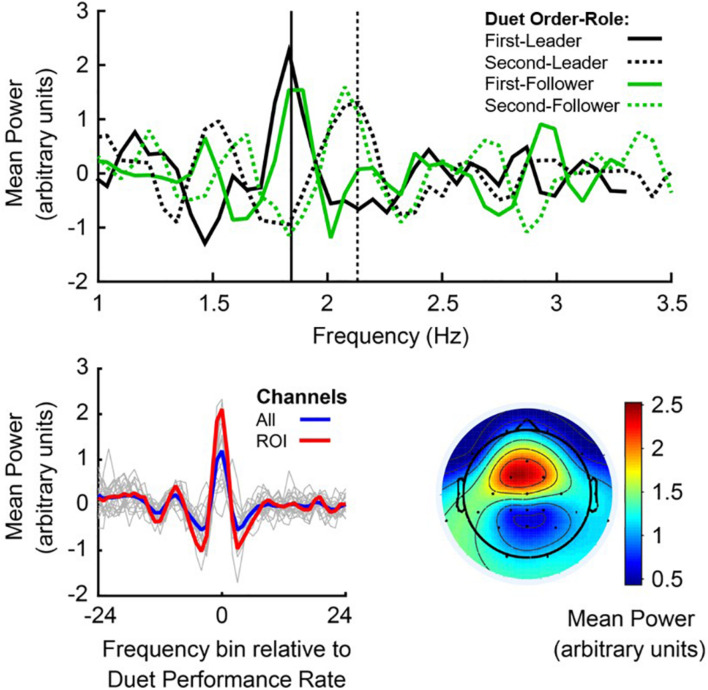
Power Spectral Density, centered at the Duet performance rate. Top panel: Noise-normalized power spectra from a sample pair of duet pianists for First-Leader (solid lines) and Second-Leader conditions (dashed lines), where the Leader’s mean Power in each condition is shown in black and the Follower’s mean Power in each condition is shown in green. Frequency (Hz) is shown on the x-axis and mean noise-normalized power is shown on the y-axis. Vertical lines indicate the mean Duet performance rate (averaged across partners) in Hertz (# tones per second) in First-Leader (solid vertical) and Second-Leader (dashed vertical) conditions. Bottom panel (left): Mean power spectra centered at the frequency of each Duet performance, shown for each pair in thin gray lines and grand average across pairs in blue; *a priori* defined auditory-motor ROI (FC1, FC2, Fz, and Cz) in red. The zero-frequency bin on the x-axis represents each pair’s Duet performance rate (center of each spectrum). Bottom panel (right): Grand average topography of spectral power at the mean Duet performance rate (zero-frequency bin of left panel), averaged within pair across conditions and then across pairs.

EEG spectral power at the Duet Performance Rate was defined for each pianist as mean PSD at the frequency bin closest to the mean Duet Performance Rate, averaged across electrode sites (channel mean PSD). Figure 4, top panel, shows mean noise-normalized spectra from members of a sample pair in each Duet condition (First-Leader condition in solid lines, Second-Leader in dashed lines), where black lines depict the Leader in each condition and green lines depict the Follower. Vertical lines indicate the frequency closest to this pair’s mean Duet Performance Rate for each condition. This figure illustrates that each partner showed a peak in spectral power at the frequency closest to the pair’s mean Duet Performance Rate in each condition, both when they were Leader and Follower (as expected, since Leaders and Followers had similar rates within performance).

To assess whether each individual pianist’s spectral power at the Duet performance rate changed as a function of their musical role (Leader/Follower), a one-way repeated-measures ANOVA on mean noise-normalized PSD at their Duet performance rate was conducted with Role (Leader/Follower) as a fixed factor and Subject as the random variable. Individual pianists’ spectral power did not change as a function of musical Role, both across channels, *F*(1,39) = 1.256, *p* = 0.269, and at the auditory-motor ROI, *F*(1,39) = 0.287, *p* = 0.595, indicating stability of spectral power within individual pianists across musical Roles.

Figure 4, bottom left, shows the mean noise-normalized spectra across channels for each pair (light gray lines). The blue line represents the grand average across channels and the red line represents the grand average at the auditory-motor ROI. The significance of this spectral peak for all duet pairs was evaluated separately for each Duet condition. Wilcoxon signed-rank tests were computed on the difference in medians between noise-normalized PSD at the frequency bin closest to the Duet Performance Rate (in Hertz) and mean PSD at the surrounding ± 8 frequency bins (John and Picton, 2000). Both Duet conditions showed statistically significant peaks in spectral power at the Duet Performance Rate relative to the mean of the neighboring frequencies (*z*_First–Leader_ = 4.50, *p* < 0.001; *z*_*Second–Leader*_ = 4.31, *p* < 0.001), confirming the prediction of increased power of neural oscillations at the Duet performance frequency in both Duet conditions.

#### PSD Peaks at Auditory-Motor ROI

Figure 4, bottom right, shows the mean topography of noise-normalized PSD at the Duet performance rate. It can be observed that power was maximal at fronto-central sites characteristically associated with auditory-motor perception and production (Nozaradan et al., 2011, 2015, 2016; Zamm et al., 2019). We evaluated whether participants showed greater neural entrainment to the Duet performance rate at these electrode sites, specifically at the ROI (section “Power Spectral Density of Oscillations at Duet Performance Rate”). To evaluate whether enhanced spectral power indicated the presence of a peak at the Duet performance rate in both conditions, ROI power at the Duet performance rate was compared with mean ROI power at the surrounding 8 frequency bins (Duet performance rate ± 8 bins). A Wilcoxon signed-rank test indicated that spectral power at the Duet performance rate was indeed higher than power at surrounding frequencies (*z*_First–Leader_ = 4.31, *p* < 0.001; *z*_*Second–Leader*_ = 5.15, *p* < 0.001), confirming the presence of a spectral peak at the Duet rate. A Wilcoxon signed-rank test indicated that ROI spectral power at the Duet rate did not differ between conditions (z = −0.07, *p* = 0.95). Figure 4, bottom (left, red line), shows the grand average noise-normalized spectrum at the ROI. A clear peak can be observed at the Duet performance rate.

### PSD at Duet Performance Rate Correlates With Duet Synchronization Accuracy

**FIGURE 5:**
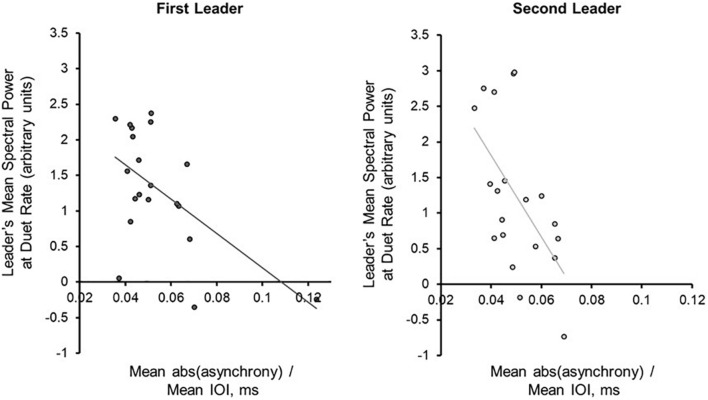
Power Spectral Density at the Duet performance rate is associated with synchronization accuracy. Correlations (with trend lines) of mean duet synchronization accuracy (absolute tone onset asynchrony divided by mean duet IOI) with mean spectral power at the Duet performance rate in the First-Leader (left) and Second-Leader (right) Duet conditions. Each dot represents one pair.

Next, the Duet pairs’ synchronization accuracy (mean absolute asynchrony divided by the mean performance beat IOI) in each condition was compared directly with mean PSD at the Duet performance rate. Synchronization accuracy and mean PSD were negatively correlated for Leaders [*r*_First–Leader_ (18) = −0.569, *p* = 0.009; *r*_Second–Leader_ (18) = −0.571, *p* = 0.009], and less strongly correlated for Followers [*r*_First–Leader_ (18) = −0.285, *p* = 0.224; *r*_Second–Leader_ (18) = −0.448, *p* = 0.048]. As shown in Figure 5, the larger the PSD values for Leaders, the smaller the Duet asynchrony. One duet pair in the First Leader condition showed a mean asynchrony value of 0.1236 (12.36% of their mean IOI), slightly greater than 3SD from the mean value for this condition ( = 0.1124). The correlation between synchronization accuracy and mean PSD in the First Leader condition recomputed without this pair was marginally significant, *r*(17) = −0.4601, *p* = 0.0845. Due to the small sample size and the fact this pair’s overall synchronization accuracy was still quite high, this pair was retained in subsequent analyses.

For each Duet condition, a multiple regression model was implemented to predict mean absolute Duet asynchrony, adjusted for Duet performance rate, from Leader and Follower PSD at the Duet performance rate. For the First-Leader condition, this model was significant, *R*^2^ = 0.33, *p* = 0.036: The Leader’s PSD was a significant predictor of Duet asynchrony, standardized β = −0.578, *t*(17) = 2.47, *p* = 0.025, whereas the Follower’s PSD was not, standardized β = 0.02, *t* (17) = 0.08, *p* = 0.94. For the Second-Leader condition, this model was also significant, *R*^2^ = 0.34, *p* = 0.03: The Leader’ PSD was a marginally significant predictor of Duet PSD, standardized β = −0.480, *t*(17) = 1.88, *p* = 0.08, whereas the Follower’s was not, standardized β = −0.14, *t*(17) = 0.56, *p* = 0.58. Thus, Leaders’ PSD values increased as the Duet partners’ asynchrony decreased, regardless of which partner served as Leader (First- or Second-Leader).

### EEG Amplitude Envelope Correlations

**FIGURE 6:**
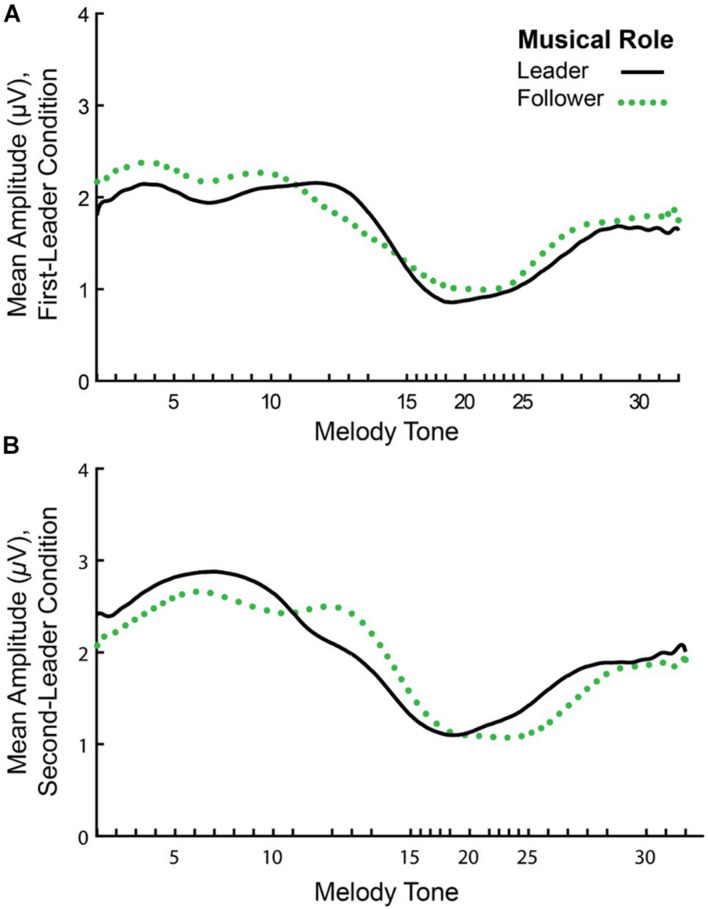
Mean amplitude envelopes of one pair’s neural oscillations at the Duet performance rate. Timecourse of amplitude fluctuations in spectral energy at the Duet performance rate for a sample pair in the First-Leader **(A)** and Second-Leader **(B)** conditions. In each condition, the Leader’s mean amplitude envelope across 24 melody performances is shown in black (solid line) and the Follower’s mean amplitude envelope is shown in green (dotted line).

Figure 6 shows the mean amplitude envelopes computed at the Duet performance rate for a sample pair (Leader/Follower) in First-Leader (Panel A) and Second-Leader (Panel B) Duet conditions. It can be observed from this figure that partners show similar amplitude fluctuations across the time series within each condition. Correspondences in partners’ amplitude envelopes were quantified by computing inter-brain Amplitude Envelope Correlations (AECs). As described in section “Inter-Brain Correlations of Duet Partners’ Amplitude Envelopes”, AECs within each condition were computed as Fisher *r-*to-*z* scores. Because Fisher *r*-to*-z* scores did not differ significantly between First-Leader and Second-Leader conditions, *F*(1, 19) = 1.83, *p* = 0.19, each Duet pair’s condition-mean Fisher *r-*to-*z* scores were averaged across conditions. This procedure yielded a single mean Fisher *r-*to-*z* score per Duet pair (*N* = 20), which was then converted to a Pearson’s *r*-value. The mean observed AEC across pairs and conditions was *r* = *0.26* (range of *r-*values across pairs = −0.04 to 0.57).

**FIGURE 7:**
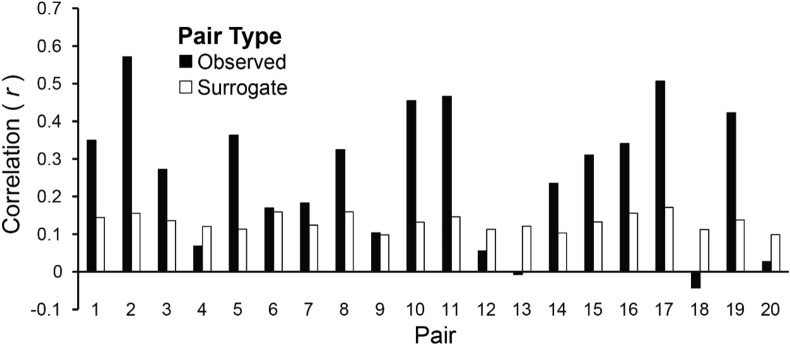
Amplitude envelopes at the Duet performance rate are correlated between duet partners. Mean correlations of duet partners’ mean amplitude fluctuations in spectral energy at the Duet performance rate are shown for each pair, averaged across Duet conditions (black bars). Pairs are ordered by difference in Solo performance rates as shown in Figure 2A (smallest difference at left, largest at right). Mean correlations of surrogate partners’ amplitude envelopes (based on 19 surrogate pairings for each partner with all other partners) are shown for each pair, averaged across Duet conditions (white bars).

Amplitude envelopes may be correlated across Duet partners because they exhibited inter-brain correspondences, or, alternatively, because they performed the same task (each Duet pair performed the same melody). To test whether the AEC values were pair-specific, the observed values were compared with the chance estimate based on mean AECs of each pair’s surrogate distribution generated from the re-pairing of data from different duet partners (described in section “Inter-Brain Correlations of Duet Partners’ Amplitude Envelopes”). Figure 7 shows the observed mean amplitude envelope correlation for each pair, and the mean correlation of each pair’s surrogate distribution, which represents the expected correlation between amplitude envelopes of pianists performing the same melody at different times. Binomial tests comparing the observed inter-individual envelope correlations with surrogate distributions indicated that the observed correlations were significantly higher than the surrogate correlations. The mean observed inter-pair correlation was higher than the mean chance (across-pair) Pearson correlation values in 15/20 pairs (*p* = 0.02; median observed *r* = 0.29, median surrogate *r* = 0.13).

## Discussion

The current study demonstrated that two pianists’ spontaneous performance rates were associated with their ability to synchronize actions when performing duets. Specifically, differences in partners’ spontaneous rates were positively correlated with Duet asynchronies: Partners with larger differences in spontaneous rates showed larger signed tone onset asynchronies. This finding is consistent with dynamical systems accounts that predict period coupling between oscillations as a function of the difference in their natural frequencies (Haken et al., 1985; Pikovsky et al., 2003). In contrast to previous studies (Schmidt and O’Brien, 1997; Richardson et al., 2005, 2007; Lopresti-Goodman et al., 2008; Loehr and Palmer, 2011; Zamm et al., 2015, 2016), the pianists’ duet performances were not paced by an external cue. Thus, this finding demonstrates non-linear dynamics in human interpersonal coordination in the context of natural, uncued joint action.

The current findings add to a growing body of work indicating that spontaneous music performance rates reflect oscillatory processes that influence auditory-motor entrainment within and between individuals (Zamm et al., 2016, 2018; Scheurich et al., 2018; Palmer et al., 2019; Roman et al., 2020). Whether the spontaneous performance rates reflect intrinsic timekeeping processes or biomechanical constraints is a topic for further research. We also observed period coupling in pianists’ neural activity during duet performance. Duet partners’ neural oscillations were enhanced at the performance frequency of produced tone onsets (Duet frequency), evidenced by peaks in each partner’s power spectral density measures at the Duet frequency. When each partner took turns being the Leader, both partners’ spectral peaks were observed at the Duet frequency determined by the Leader. Moreover, enhanced power at the Duet frequency was positively associated with partners’ synchronization accuracy, with stronger correlations for Leaders than for Followers. Partners with high synchronization accuracy, measured as smaller tone onset asynchronies, showed higher spectral peaks at the Duet frequency, relative to partners with low synchronization accuracy. Furthermore, synchronization accuracy was better predicted by the Leader’s spectral energy associated with the performance rate than by the Follower’s spectral energy, suggesting that the spectral peaks at performance frequencies serve as a possible marker of leadership behavior. Furthermore, synchronization accuracy was better predicted by the Leader’s spectral energy associated with the performance rate than by the Follower’s spectral energy, suggesting that the spectral peaks at performance frequencies serve as a possible marker of leadership behavior. Together, these findings demonstrate a clear link between period coupling seen in interpersonal synchrony measures and in neural oscillations at the frequency of a jointly produced action. One interesting question for future research is what determines inter-subject variability in this overall relationship, and what factors—such as entrainment at higher rhythmic groupings—may allow partners to achieve high synchrony even with low spectral energy at the common frequency of a joint action.

The observed oscillatory cortical activity in the current study may have been influenced by evoked potentials elicited by the perception of rhythmically occurring tone onsets, as suggested by an ongoing debate in the literature over whether rhythmic brain activity reflects purely oscillatory processes or also stimulus-evoked potentials elicited by rhythmic stimuli (see Haegens and Golumbic, 2018 for a review). Although the current design cannot disentangle these potential contributions to the observed cortical activity at the frequency of pianists’ performance rates, it should be noted that cortical activity was measured at the mean musical beat frequency, which does not directly necessarily correspond to the frequency at which all tone onsets occur, but rather to the perceived frequency at which tone onsets are grouped (Grahn and Brett, 2007). There is compelling evidence that beat-related brain activity is not purely stimulus-driven, but rather arises from endogenous oscillatory activity; specifically, enhanced spectral power of cortical activity can be observed at the beat frequency that subjects perceive tones to occur at, even when the tones occur at a different frequency (Nozaradan et al., 2011; Fujioka et al., 2015). Thus, enhanced cortical activity at the beat frequency of pianists’ performances in the current study likely reflects a combination of endogenous oscillatory processes and stimulus-driven contributions; an important future direction is to design studies that can disentangle these possibilities.

Duet partners also showed temporally correlated fluctuations in amplitude dynamics across the entire performance. Amplitude coupling was observed in the inter-brain correlations of partners’ spectral energy at the Duet frequency (rate of performance). Correlations between observed partners’ amplitude envelopes were higher on average than correlations between duet pianists who performed the same task but with other partners (surrogate pairs), suggesting that inter-brain amplitude coupling is partner-specific and may arise from the temporally distinctive auditory-motor patterns of each performance. It cannot be ruled out that some amount of inter-brain amplitude coupling in the current study arose from partners’ perception of musical sequences with identical rhythmic (temporal) properties. Our previous work (Zamm et al., 2018) indeed suggests that amplitude envelopes reflect the unique timing of pianists’ performances of the same categorical rhythms, and therefore it is likely that the current inter-brain correlations reflect the specific (unique) way in which each pair produced the notated stimulus rhythm. Moreover, some evidence from functional magnetic resonance imaging suggests that individuals who are independently exposed to temporally identical stimuli show inter-brain similarities in sensory responses (Hasson et al., 2004; Wilson et al., 2007; Hanson et al., 2009). Further work is needed to disentangle the extent to which inter-brain synchrony arises from such purely stimulus-driven mechanisms versus interpersonal joint action. Finally, it should be noted that although the correlations for observed pairs were larger overall than that of surrogate pairs, 5 of 20 pairs showed lower amplitude correlations for observed relative to surrogate pairings; these pairs also showed lower envelope correlations than other pairs, suggesting that possibly these pairs did not display clear amplitude tracking of the musical rhythm. The current envelopes were extracted from spatial filters representing a mix of auditory, motor, and other sources; it is possible that the ability to detect clear amplitude modulations at the beat frequency differs for participants whose entrainment arose from more than one source. An open question for future research is to identify factors that determine individual differences in amplitude envelope coupling.

The current study demonstrates the novel application of amplitude envelope correlations to inter-brain correspondences. Previously used to detect functional connectivity or coupling *within* individuals’ brain networks (Bruns et al., 2000; Doron et al., 2012; Hipp et al., 2012), we applied AECs to detect coupling *between* individuals. Although the current findings are agnostic with respect to the relationship between phase-resetting mechanisms of endogenous neural oscillations and transient ERPs in response to rhythmic auditory stimuli (Haegens and Golumbic, 2018; Novembre and Iannetti, 2018), the measures of enhanced power and amplitude envelopes presented here offer insights into the bidirectional (Leader-Follower) period coupling typical of skilled musical ensembles (Demos et al., 2019).

A remaining question is how these amplitude-based metrics of inter-brain correspondences compare with alternative measures of inter-brain synchrony, such as phase-based metrics. Some work suggests that amplitude and phase are related (Canolty et al., 2006; Cohen et al., 2008; Lee and Jeong, 2013; Combrisson et al., 2017); however, amplitude coupling metrics may capture correspondences in cortical oscillations that may not be detected by phase-based metrics (Bruns et al., 2000), and may show greater test-retest reliability (Colclough et al., 2016). Future work should investigate how amplitude- and phase-based metrics of inter-brain correspondences are related, and whether partners with similar natural frequencies of music performance show enhanced inter-brain phase-locking relative to partners with different natural frequencies.

Finally, the current study implements what is to our knowledge the first simultaneous recording of wireless EEG from performing ensemble musicians. Wireless EEG with head-mounted amplifiers enables individual to walk freely, with modest motion artifact (Debener et al., 2012; De Vos et al., 2014a, b). However, movement artifacts due to free walking can confound brain activity (Jacobsen et al., 2020). Although pianists in the current study remained seated and may not have moved as much as during a natural concert performance featuring expressive body movement, or free walking, wireless EEG has been shown to successfully measure brain activity in numerous highly active contexts from motor rehabilitation (Zich et al., 2015) to speech production (Fjaellingsdal et al., 2016) to bike riding (Scanlon et al., 2019) and memory encoding (Piñeyro Salvidegoitia et al., 2019), providing compelling evidence that brain activity can clearly be captured using wireless EEG even under high activity loads. We thus propose that two wireless EEG amplifiers can capture the coupling dynamics associated with temporal coordination between expressively performing musicians. Future studies may include motion sensors attached to different body parts to explore whether movement artifacts compromise measure of neural interpersonal synchrony. This presents new possibilities for measuring the neural correlates of interpersonal coordination without the motion constraints of traditional EEG. Wireless EEG could be used not only in musical duets but in larger groups such as string quartets and even orchestras, where expressive body gestures may be even more important for communication between performers (Davidson, 2012; Glowinski et al., 2013; Badino et al., 2014). We hope that these findings set a precedent for more ecological measurement of oscillator dynamics between individuals acting together.

## Data Availability Statement

The raw data and code supporting the conclusions of this article will be made available by the authors upon reasonable request, without undue reservation.

## Ethics Statement

The studies involving human participants were reviewed and approved by Kommission für Forschungsfolgenabschätzung und Ethik. The participants provided their written informed consent to participate in this study.

## Author Contributions

AZ designed the experiment in collaboration with co-authors, conducted the experiment, implemented and interpreted analyses, wrote drafts of the manuscript and incorporated the revisions. CP contributed to and provided supervision in experiment design, supervised implementation of data analysis, contributed to interpreting results and assisted in writing and revising the manuscript for publication. A-KRB contributed to experiment design, gave input on implementation of EEG data analyses, and assisted with data interpretation and manuscript revision. MGB contributed to experiment design and technical set-up, gave input on implementation of EEG data analyses, and assisted with data interpretation and manuscript revision. APD gave input on statistical analyses of data, and assisted with manuscript revision. SD supervised and contributed to experiment design and implementation, provided laboratory space and mobile EEG equipment for data collection, supervised implementation of data analysis, and contributed to interpreting results, assisted in writing and revising the manuscript for publication. All authors contributed to the article and approved the submitted version.

## Conflict of Interest

The authors declare that the research was conducted in the absence of any commercial or financial relationships that could be construed as a potential conflict of interest.

## Publisher’s Note

All claims expressed in this article are solely those of the authors and do not necessarily represent those of their affiliated organizations, or those of the publisher, the editors and the reviewers. Any product that may be evaluated in this article, or claim that may be made by its manufacturer, is not guaranteed or endorsed by the publisher.

## Footnotes

1
https://github.com/sccn/labstreaminglayer/tree/master/LSL/liblsl/

2
https://github.com/sccn/labstreaminglayer/wiki/LabRecorder.wiki

3
http://www.mbraintrain.com/smarting/

4
www.easycap.de
